# The pathway analysis of relationship between illness uncertainty, perceived stress, coping strategies, and quality of life in patients with coronary artery disease

**DOI:** 10.1371/journal.pone.0357910

**Published:** 2026-09-15

**Authors:** Atefeh Allahbakhshian, Seyyede Leila Sajjadi, Parvin Sarbakhsh, Hadiseh Tabrizi

**Affiliations:** 1 Department of Medical Surgical Nursing, Faculty of Nursing and Midwifery, Tabriz University of Medical Sciences, Tabriz, Iran; 2 Ph.D Candidate in Nursing, Student Research Committee, Tabriz University of Medical Sciences, Tabriz, Iran; 3 Health and Environmental Research Center, Tabriz University of Medical Sciences, Tabriz, Iran; Alexandria University Faculty of Nursing, EGYPT

## Abstract

**Background:**

Previous studies suggests that illness uncertainty is associated with quality of life in chronic illness, yet the underlying mechanisms remain unclear, particularly in coronary artery disease (CAD). This study aims to explore the relationship between uncertainty in illness, perceived stress, coping strategies, and quality of life in patients with coronary artery disease.

**Methods:**

This cross-sectional study was conducted in 150 patients with CAD recruited at a specialized heart clinic affiliated with Tabriz University of Medical Sciences, Tabriz (Iran) in 2024. Uncertainty in illness, perceived stress, coping strategies, and QoL were evaluated through self-reported questionnaires. The multiple mediation model was analyzed using SPSS and Amos software, with a P-value of less than 0.05 considered significant.

**Results:**

The mean (SD) scores of uncertainty in illness, perceived stress, coping strategies, physical, and psychological quality of life were 66.77 (11.97) (in a range of 23–115), 29.57 (4.96), 59.91 (10.32), 11.95 (2.90), and 16.48 (1.69), respectively. The Pearson correlation indicated a significant positive correlation between uncertainty in illness and perceived stress (r = 0.50, P < 0.001). Additionally, uncertainty in illness was negatively and significantly correlated with coping strategies (r = −0.41, P < 0.001) and physical QoL (r = −0.32, P < 0.001). There was also a significant negative correlation between perceived stress and coping strategies (r = −0.29, P < 0.001) as well as between perceived stress and physical QoL (r = −0.37, P < 0.001). Furthermore, a significant positive correlation was found between psychological QoL and physical QoL (r = 0.39, P < 0.001).

**Conclusions:**

Uncertainty in illness and perceived stress are important factors that affect QoL in patients with coronary artery disease. It is recommended to implement well-structured interventions aimed at decreasing illness uncertainty and perceived stress while improving physical quality of life in patients with coronary artery disease.

## Introduction

Coronary artery disease (CAD) is a type of atherosclerotic disease that occurs with symptoms of stable angina, unstable angina, myocardial infarction, or even sudden death [1], [2]. This disease is the most common cause of death all over the world [3] and is one of the main causes of disability [4], [5]. Countries with robust preventive strategies and public awareness campaigns tend to report lower CAD incidences compared to Iran [6]. A study involving 2,000 participants over nearly 10 years reported a CAD incidence of 14.5% in Iran [6].

CAD reduces the quality of life (QoL) of patients by causing physical problems, psychological challenges, drug side effects, and social restrictions, so the QoL in patients with CAD is lower compared to the healthy population [7], [8]. A study found that 56.2% of CAD patients had very severe physical limitations, correlating significantly with lower QoL scores [9]. Furthermore, poor QoL exacerbates hospitalization and mortality in patients with heart disease [10], [11]. Therefore, exploring potential mechanisms affecting QoL in patients with CAD is necessary.

Prior researchers have shown that psychological factors like depression and anxiety are linked to QoL [12], [13]. Another important psychological factor may be uncertainty, defined as the “inability to determine the meaning of events related to the illness or the inability to predict the events of the illness.” [14].

People with chronic diseases experience uncertainty in illness due to the long duration of the disease, receiving complex treatments, reduced physical performance and other factors [15], [16]. According to Mishel’s definition, uncertainty in illness has 4 forms of uncertainty about disease stage, complexity of treatment and care system, lack of information in the field of diagnosis and the deterioration of the disease, and unpredictability of the illness process and prognosis [14].

Uncertainty in illness has a dynamic nature and can change over time [17]. Individuals try to reduce uncertainty in illness by understanding the symptoms related to their condition. Healthcare professionals can play a supportive role in this process [14]. According to Han, providing proper knowledge and information is the optimal approach to helping reduce patient uncertainty regarding their medical options [18].

Uncertainty is the main factor of stress for patients [19], [20], which leads to aggravation of psychological problems such as depression, anxiety, negative coping strategies, and reduced self-care and life satisfaction [21–23]. When patients perceive uncertainty as a risk for psychological stress, wrong decision-making increases [24], and their QoL can be impaired by decreasing adherence to treatment and decreasing coping ability [25].

Recent advances in medical therapy have shown that atherosclerotic plaque progression can be stabilized and even reversed, leading to improved cardiac function and prognosis [26]. Cesaro et al. (2024) emphasized that such therapies not only reduce cardiovascular risk but may also alleviate illness-related stress by enhancing patients’ perceptions of control and recovery potential [26]. These improvements in physiological health may translate into better psychological well-being and quality of life, underscoring the importance of addressing both biological and psychosocial dimensions in CAD care.

Based on Mishel’s uncertainty in illness theory and prior studies, the first hypothesis is that uncertainty in illness may indirectly affect QoL through perceived stress in patients with CAD. On the other hand, when patients identify uncertainty as an opportunity or a risk, they use different coping strategies [27]. Coping is the process of “continuous changing cognitive and behavioral efforts to manage specific demands.” Patients use coping strategies to manage the disease, which includes 3 main strategies: exposure, avoidance, and acceptance [28]. Coping strategies have a dynamic nature and individuals’ strategies for dealing with illness or stress evolve over time as their situation changes. This evolution can be shaped by the progression of the illness, the impact of the illness on their life, and the person’s psychological, social, and emotional adjustments [29]. Nahlen Bose et al found that avoidant coping strategies were linked to poorer QoL in patients with heart disease [30]. So, we propose the second hypothesis that uncertainty in illness may indirectly influence QoL through coping strategies in patients with CAD.

**Fig 1:**
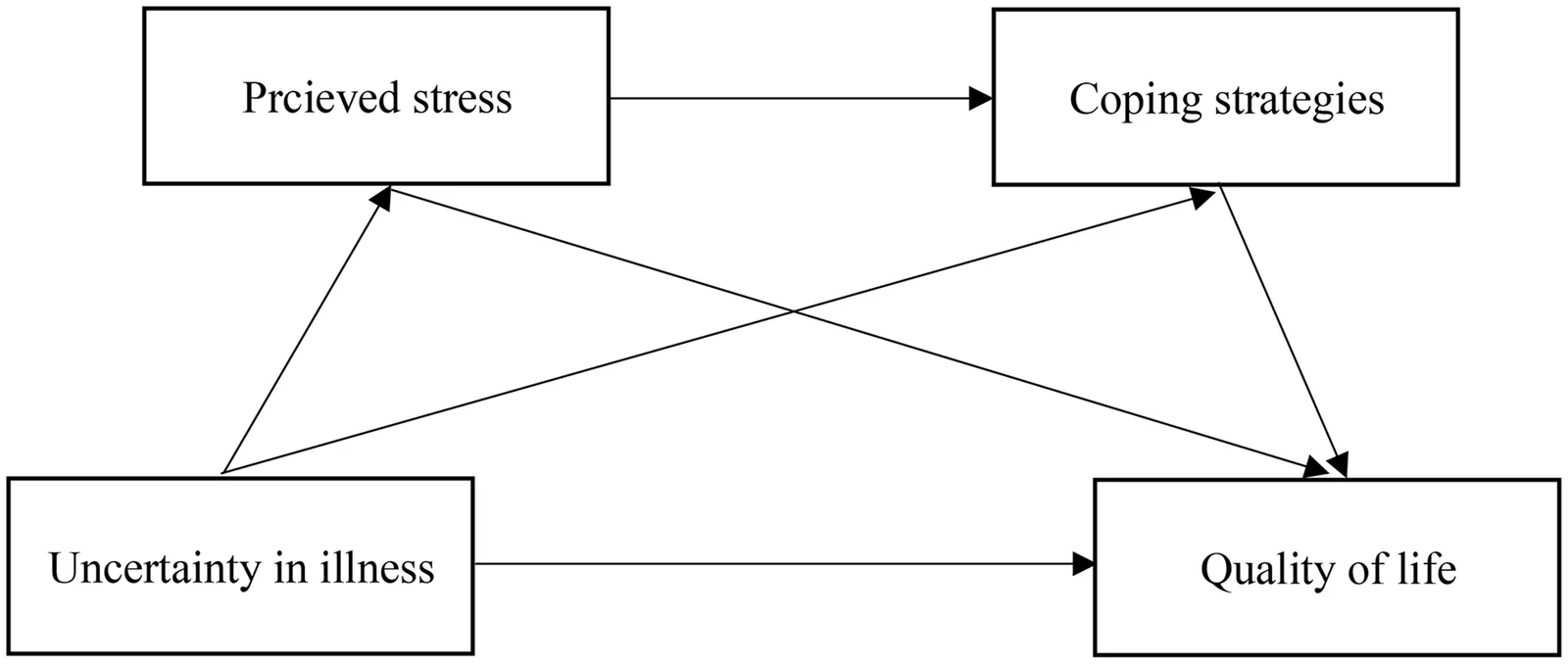
The hypothetical mediation model of the relationship between uncertainty in illness and quality of life in patients with coronary artery disease.

Based on the stress and coping theory of Lazarus and Folkman and based on the studies on the relationship between stress and physical and psychological health, it seems that coping strategies play a mediating role in this process [31]. The results of a study show that uncertainty about the future affects psychological distress and coping [32]. The results of the study by Alhurani et al. showed that avoidant emotional coping is associated with high stress and predicts the survival rate of patients with heart disease [33]. Based on the theory of stress adaptation and adaptation by Lazarus and Folkman [31] and based on previous studies, the third hypothesis is that perceived stress and coping strategies are the serial mediators in the relationship between uncertainty in illness and QoL. Fig 1 shows the hypothetical model.

This study uniquely targets patients with CAD in Iran, a demographic that may experience distinct cultural, social, and economic factors affecting their health outcomes. Previous studies have often generalized findings across various conditions or populations, which may not fully capture the nuances of this specific group. By employing pathway analysis, this study aims to explore complex and multifaceted relationships between the variables involved. This method allows for a comprehensive understanding of how illness uncertainty and perceived stress influence coping strategies and ultimately affect the QoL, providing insights that simpler analytical methods may overlook. The findings from this study will provide localized insights that can guide healthcare professionals in Iran, allowing them to develop tailored interventions that cater to the specific needs and challenges faced by patients with CAD in the region.

## Materials and methods

### Aim/ objectives

This study aims to assess the relationship between uncertainty in illness, perceived stress, coping strategies, and QoL in patients with CAD.

### Study design and setting, and sample size

This correlational cross-sectional study was conducted at a specialized heart clinic affiliated with Tabriz University of Medical Sciences, Tabriz (Iran), from July 2024 to December 2024. Patients were recruited consecutively as they attended outpatient follow-up visits or were hospitalized for CAD management during the study period to minimize selection bias. All eligible patients who met the inclusion criteria and provided informed consent were invited to participate.

The inclusion criteria included 1. Patients with a definitive diagnosis of CAD based on the diagnosis of a cardiologist; 2. Patients who have been diagnosed for at least three months; 3. Patients aged 18 and older; 4. The ability to communicate and the absence of visual, hearing, and speech disorders; 5. Absence of psychiatric disorders according to the patient’s medical record; 6. Not having a malignant or incurable disease based on the Charlson Comorbidity Index [34]; 7. Absence of cognitive disorders based on the mini-mental state examination (MMSE) scale [35]; 8. Having the ability to understand and complete questionnaires; 9. Willingness to participate in the study. The exclusion criteria included 1) unwillingness to continue participation in the study; 2) The occurrence of unexpected problems for the patient, such as the occurrence of angina pains.

Considering the existence of about 10 unknown parameters in the estimation model and the recommendation of 10–20 samples for each parameter [36]15 samples for each parameter were considered in this study. The final sample size is estimated at 150 patients. The convenience sampling method was used for data collection.

### Data collection measures

The data in this study were collected using demographic information, Mishel’s uncertainty in illness scale – Community Form (MUIS-C), perceived stress scale, Endler & Parker’s coping strategies scale, and Short Form Health Survey (SF-12), using face-to-face interviewing and using patients’ medical records. The interview was conducted at the bedside of each patient. Written informed consent was obtained from all participants, and the research method was conducted through the Helsinki Declaration.

**The demographic information questionnaire:**The variables encompassed participants’ age, gender, education level, marital status, residence, living with, occupation, income, and comorbidities. illness duration, and habitual history.

**The mini-mental state examination (MMSE):** The MMSE is the most widely used cognitive assessment. It comprises 19 subtests across 11 domains, including orientation, registration, attention or calculation (such as serial sevens or spelling), recall, naming, repetition, comprehension (both verbal and written), writing, and construction. The total possible score ranges from 0 to 30 [35]. Thresholds for cognitive impairment vary with the studied population. For the general Iranian population, prior studies indicate that a Persian MMSE score below 23 suggests potential cognitive decline [37]. MMSE was administered by trained staff at screening.

**Uncertainty in illness:** Mishel’s Uncertainty in Illness Scale – Community Form (MUIS-C) was used to measure uncertainty [34]. This scale consists of 23 items rated on a Likert scale from 1 to 5, where 1 means strongly disagree (score = 1), 2 means disagree (score = 2), 3 means undecided (score = 3), 4 means agree (score = 4), and 5 means strongly agree (score = 5). Items 6, 8, 19, 20, 22, and 23 are scored in reverse. The scores from all items are summed, with a higher total score indicating greater uncertainty [34]. Bailey et al. demonstrated the construct validity of this scale, reporting a Cronbach’s α of 0.85 from a sample of 1,068 chronically ill adults [38]. Sajjadi et al. (2025) evaluated the Persian version of the scale and found a Cronbach’s alpha of 0.72 in Iran [17]. In the present study, Cronbach’s alpha was 0.78, indicating satisfactory reliability among CAD patients.

**Perceived stress scale:** the perceived stress scale designed by Cohen et al. (1983) was used [39]. The questionnaire contains 14 items on a 5-point Likert scale from 0 (never) to 4 (all the time). The total score ranges from 0 to 56. A score below 28 indicates low perceived stress, while a score of 28 or higher signifies high perceived stress. The reliability of the Persian version of the questionnaire was reported as 0.84 by Cronbach’s alpha method in Asghari et al.’s study [40]. In this study, the validity of the Persian versions of the perceived stress scale was assessed through content validity. The forward-backward translation method was used for the translation of the questionnaires. Then, the questionnaire was given to ten professors from the Faculty of Nursing and Midwifery to provide their comments on the content of the tools. The necessary revisions were done based on their feedback and comments. The Cronbach’s alpha for the perceived stress scale was obtained as 0.89 in our study, showing an acceptable level of reliability.

**Coping strategies scale:** This scale was designed by Endler & Parker in 1999 [41]. It includes 21 items and three subscales of problem-oriented coping, emotion-oriented coping, and avoidant coping. The score of each item consists of a five-point Likert scale from 1 “never” to 5 “always.” The total score varies from 21 to 105. The internal consistency coefficient was reported as 0.92 in previous studies [41]. The validity of the Persian version of the scale has been examined by Ghoreyshi Rad in 2010. She reported a Cronbach’s alpha coefficient of 93% for the scale in Iran [42]. In our sample, Cronbach’s α was 0.92, confirming strong internal consistency for CAD patients.

**Quality of life:** In order to evaluate the QoL, the Short Form Health Survey (SF-12)) was used. This tool was designed by Ware et al. in 1996 [38]. It includes dimensions of physical and psychological QoL. The scores for the physical and psychological dimensions range from 6 to 20, 6–28, and the total score ranges from 12 to 48. A higher score indicates a higher QoL. A score between 12 and 24 indicates low QoL, 25–36 reflects an average QoL, and 37–48 corresponds to a good QoL. The test-retest reliability of this scale is reported as 0.89 and 0.76 for the physical and psychological dimensions, respectively by Ware et al.. Also, Montazeri et al. in Iran reported the reliability of the physical and psychological dimensions as 0.73 and 0.72, respectively ((72)). The Persian version used in this study demonstrated reliability coefficients of 0.83 for physical and 0.79 for psychological subscales, consistent with previous studies in Iranian cardiac patients.

### Data analysis

Data were processed, coded, and analyzed using Amos and SPSS software, version 26. Descriptive statistics, such as frequency, mean, percentage, and standard deviation (SD) were used to summarize the participants’ characteristics, their uncertainty in illness, perceived stress, coping strategies, and QoL. Pearson’s correlation analysis was used to examine the association between uncertainty in illness, perceived stress, coping strategies, and QoL. The level of statistical significance was set at p < 0.05.

In addition to examining the relationships between illness uncertainty, coping strategies, perceived stress, and quality of life, path analysis was conducted to test the hypothesized model and to examine both direct and indirect (mediation) relationships among variables based on the theoretical framework. The model parameters were estimated using maximum likelihood estimation. Prior to analysis, assumptions including normality and absence of multicollinearity were assessed.

Model fit was evaluated using multiple indices, including the chi-square to degrees of freedom ratio (χ²/df), comparative fit index (CFI), incremental fit index (IFI), non-normed fit index (NNFI/TLI), root mean square error of approximation (RMSEA), and standardized root mean squared residual (SRMR). Values of CFI, IFI, and NNFI ≥ 0.90 indicate acceptable fit, while RMSEA and SRMR values ≤ 0.08 indicate good model fit.

Given the cross-sectional design of the study, the proposed model reflects statistical associations rather than causal relationships; therefore, causal interpretations should be made with caution. The structural model showed an acceptable overall fit to the data, with χ²(2)=3.698 (p = 0.157), χ²/df = 1.85, CFI = 0.988, TLI (NNFI)=0.889, RMSEA = 0.078, and SRMR = 0.024. The non-significant chi-square statistic indicates that the hypothesized model did not significantly differ from the observed data. Although the TLI value was slightly below the conventional cutoff of 0.90, the CFI, SRMR, and RMSEA values collectively support an acceptable fit of the proposed structural model.

### Ethics approval and consent to participate

Ethics approval was obtained from the Research Ethics Committee of Tabriz University of Medical Sciences (Code: IR.TBZMED.REC.1402.227). Written informed consent was obtained from all participants and the research method was conducted through the Helsinki Declaration.

## Results

### Patient characteristics

The demographics and clinical characteristics of the 150 patients are summarized in Table 1. From 150 participants, half of them were female (50%) and most of them were married (84.66%). 54% had a Diploma education level and 49.3% were employee. More than half of the patients had Income less than expenses (62.66%) and were living in the city (93.33%). The mean (SD) age of the patients was 48.58 (11.21) years.

**Table 1:** Comparison of QoL by Sample Characteristics (N = 150).

Variables	Categories	N (%)	Physical QoL			Psychological QoL		
			Mean (SD)	t/F	P	Mean (SD)	t/F	P
Gender	Male	75 (50.00)	11.60 (2.92)	2.44	0.09	16.47 (1.51)	0.05	0.94
	Female	75 (50.00)	12.22 (2.78)			16.51 (1.85)		
Education level	Illiterate	9 (6.00)	15.00 (2.82)	12.87	<0.001	17.00 (1.41)	4.09	0.008
	Less than Diploma	32 (21.33)	10.10 (2.80)			15.55 (1.82)		
	Diploma	81 (5.004)	11.69 (2.47)			16.62 (1.37)		
	University	28 (18.66)	13.94 (2.71)			16.88 (2.02)		
Marital status	Married	127 (84.66)	11.83 (2.77)	4.06	0.01	16.40 (1.72)	1.22	0.29
	Single	14 (9.30)	13.92 (3.66)			17.00 (1.70)		
	Divorced/ Widowed	9 (6.00)	11.00 (1.73)			17.00 (0.86)		
residence	City	140 (93.33)	12.03 (2.84)	3.06	0.08	16.50 (1.70)	0.54	0.46
	Village	10 (6.77)	10.40 (3.06)			16.00 (1.44)		
Living with	With spouse	123 (82.00)	11.81 (2.77)	6.41	0.002	16.39 (1.74)	1.23	0.29
	With parents	10 (6.70)	14.90 (2.13)			17.20 (1.03)		
	Lonely	15 (10.00)	11.13 (3.20)			16.73 (1.57)		
Occupation	Employee	74 (49.30)	11.98 (3.13)	0.67	0.57	16.47 (1.65)	0.58	0.62
	House wife	52 (34.70)	11.80 (2.55)			16.40 (1.77)		
	Retired	19 (11.30)	12.41 (2.76)			16.94 (1.39)		
	Unemployed	5 (3.30)	10.40 (2.19)			16.00 (2.44)		
Income	Income is less than expenses	94 (62.66)	11.53 (2.59)	3.61	0.03	16.46 (1.67)	0.06	0.93
	Income equals expenses	51 (34.00)	12.68 (3.28)			16.56 (1.72)		
	Income is more than expenses	5 (3.33)	13.80 (2.58)			16.40 (2.07)		
Hypertension History	Yes	125 (83.33)	11.89 (2.83)	0.29	0.58	16.58 (1.55)	0.26	0.60
	No	25 (16.77)	12.24 (3.15)			16.40 (1.87)		
Diabetes History	Yes	32 (21.33)	11.03 (3.15)	3.53	0.06	16.31 (1.59)	0.91	0.34
	No	118 (78.66)	12.10 (2.72)			16.61 (1.57)		
Dyslipidemia History	Yes	45 (30.00)	10.82 (2.60)	11.14	0.001	16.57 (1.51)	0.004	0.95
	No	105 (70.00)	12.43 (2.68)			16.56 (1.58)		
Time since diagnosis	6-12 months	34 (22.66)	13.28 (2.79)	5.24	0.002	16.75 (1.56)	0.43	0.72
	1-2 years	38 (25.33)	12.38 (2.73)			16.30 (1.75)		
	2-4 years	39 (26.00)	11.08 (2.53)			16.56 (1.51)		
	More than 4 years	39 (26.00)	11.05 (3.03)			16.44 (1.76)		
Doing exercise	Yes	18 (12.00)	12.94 (3.22)	2.65	0.07	16.88 (1.71)	3.06	0.05
	No	94 (62.55)	11.55 (2.60)			16.22 (1.64)		
	Sometimes	38 (25.33)	12.50 (3.29)			16.94 (1.68)		
Angiography History	Yes	36 (24.00)	9.83 (2.59)	30.02	<0.001	16.19 (1.63)	1.32	0.25
	No	114 (76.00)	12.60 (2.65)			16.56 (1.70)		
Habitual history	Yes	52 (34.77)	11.88 (2.99)	0.04	0.83	16.61 (1.41)	0.44	0.50
	No	98 (65.33)	11.98 (2.84)			16.42 (1.81)		

SD = Standard deviation, QoL = Quality of life

### Mean scores and correlation coefficients of study variables

Comparisons of the physical and psychological QoL using the sample characteristics are presented in Table 1. Poorer physical and psychological QoL was found among patients who had less than a Diploma education level (P = 0.008). Patients who were single, living with parents, had income exceeding expenses, lacked a history of dyslipidemia, and experienced a shorter illness duration demonstrated an improved physical QoL.

**Fig 2:**
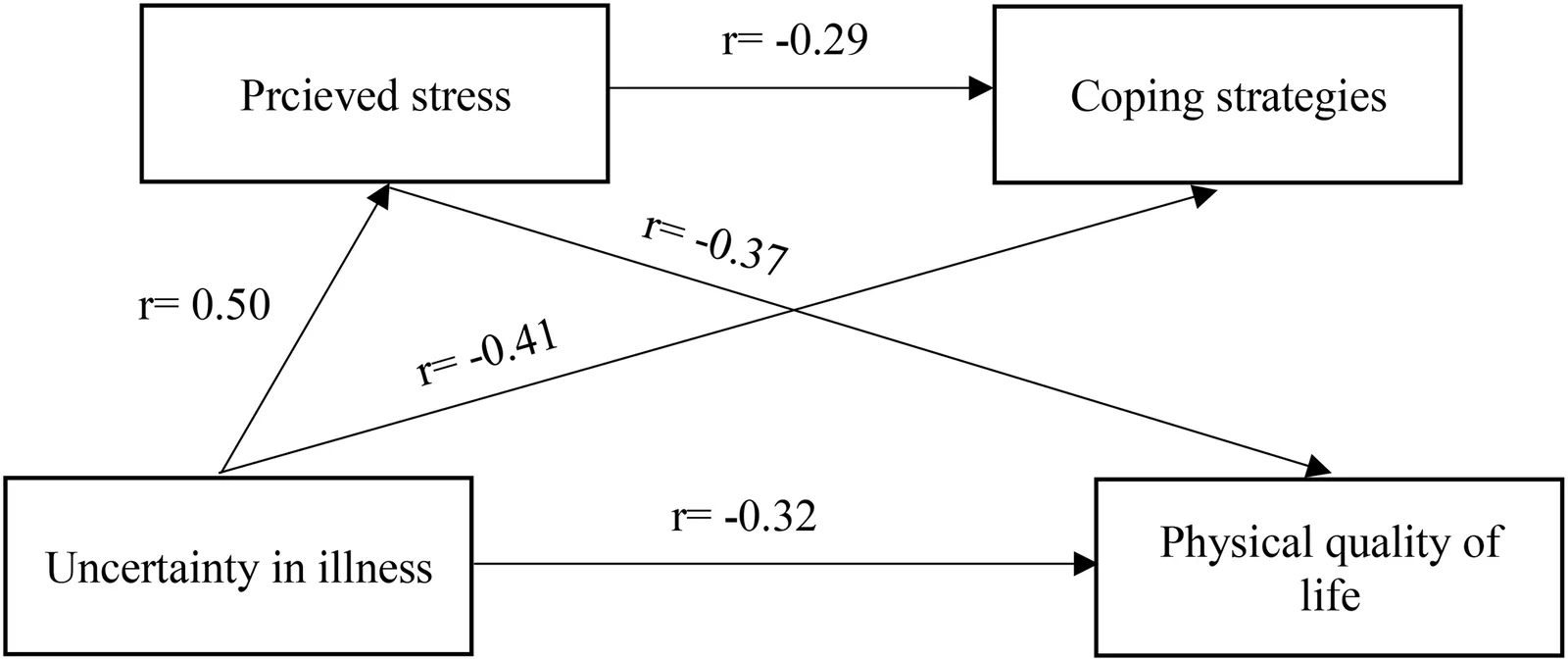
The relationship between uncertainty in illness, perceived stress, coping strategies, and quality of life in patients with coronary artery disease.

Mean scores and correlation coefficients of uncertainty in illness, perceived stress, coping strategies, and physical and psychological QoL are shown in Table 2 and Fig 2. Mean (SD) physical and psychological scores were 11.95 (2.90) (in a range of 6–20) and 16.48 (1.69) (in a range of 6–28). Also, the mean scores of uncertainty in illness, perceived stress, and coping strategies were 66.77 (11.97) (in a range of 23–115), 29.57 (4.96) (in a range of 0–56), and 59.91 (10.32) (in a range of 21–105), respectively.

**Table 2:** Mean scores and correlation coefficients of the QoL, uncertainty in illness, perceived stress, and coping strategies (N = 150).

	Mean (SD)	Uncertainty	Perceived stress	Coping strategies	Physical QoL	Psychological QoL
Uncertainty	66.77 (11.97)	1				
Perceived stress	29.57 (4.96)	0.50 **P < 0.001**	1			
Coping strategies	59.91 (10.32)	−0.41 **P < 0.001**	−0.29 **P < 0.001**	1		
Physical QoL	11.95 (2.90)	−0.32 **P < 0.001**	−0.37 **P < 0.001**	0.05 P = 0.47	1	
Psychological QoL	16.48 (1.69)	−0.04 P = 0.59	−0.12 P = 0.11	−0.03 P = 0.70	0.39 **P < 0.001**	1

SD = Standard deviation, QoL = Quality of life

The Pearson correlation indicated a significant positive correlation between uncertainty in illness and perceived stress (r = 0.50, P < 0.001). Additionally, uncertainty in illness was negatively and significantly correlated with coping strategies (r = −0.41, P < 0.001) and physical QoL (r = −0.32, P < 0.001). There was also a significant negative correlation between perceived stress and coping strategies (r = −0.29, P < 0.001) as well as between perceived stress and physical QoL (r = −0.37, P < 0.001). Furthermore, a significant positive correlation was found between psychological QoL and physical QoL (r = 0.39, P < 0.001) (Table 2, Fig 2).

Table 3 presents the direct, indirect, and total effects among the study variables.

**Table 3:** Multiple mediating effects test of perceived stress and coping strategies between uncertainty in illness and QoL (N = 150).

Dependent variable	Independent variable	Statistical values				
		Effect	SE	P-value	Std. coef	95% CI
Uncertainty in Illness	Age					
	-Direct effect	0.04	0.09	0.66	0.03	0.13 to 0.21
	-Indirect effect	No path				
	-Total effect	0.04	0.09	0.66	0.03	0.13 to 0.21
	Education level					
	-Direct effect	−1.10	0.98	0.26	−0.09	−3.02 to 0.82
	-Indirect effect	No path				
	-Total effect	−1.10	0.98	0.26	−0.09	−3.02 to 0.82
	Income					
	-Direct effect	−6.81	1.81	0.00	−0.31	−10.35 to −3.27
	-Indirect effect	No path				
	-Total effect	−6.81	1.81	0.00	−0.31	−10.35 to −3.27
Perceived stress	Uncertainty in Illness					
	-Direct effect	0.21	0.02	<0.001	0.50	0.17 to 0.24
	-Indirect effect	No path				
	-Total effect	0.21	0.02	<0.001	0.50	0.17 to 0.24
	Age					
	-Direct effect	−0.00	0.03	0.77	−0.02	−0.05 to 0.05
	-Indirect effect	0.00	0.02	0.66	0.02	−0.03 to 0.03
	-Total effect	−0.00	0.04	0.98	−0.00	−0.07 to 0.07
	Education level					
	-Direct effect	−0.36	0.36	0.31	−0.07	−1.06 to 0.34
	-Indirect effect	−0.24	0.21	0.26	−0.05	−0.65 to 0.17
	-Total effect	−0.61	0.42	0.15	−0.12	−1.43 to 0.21
	Income					
	-Direct effect	No path				
	-Indirect effect	−1.50	0.45	0.001	−0.17	−4.34 to 1.34
	-Total effect	−1.50	0.45	0.001	−0.17	−4.34 to 1.34
Coping strategies	Perceived Stress					
	-Direct effect	−0.21	0.17	0.21	−0.10	−0.54 to 0.12
	-Indirect effect	No path				
	-Total effect	−0.21	0.17	0.21	−0.10	−0.54 to 0.12
	Uncertainty in Illness					
	-Direct effect	−0.31	0.07	<0.001	−0.36	−0.44 to −0.18
	-Indirect effect	−0.04	0.03	0.22	−0.05	−0.09 to 0.01
	-Total effect	−0.35	0.06	<0.001	−0.41	−0.46 to −0.24
	Age					
	-Direct effect	No path				
	-Indirect effect	−0.01	0.03	0.68	−0.01	−0.06 to 0.04
	-Total effect	−0.01	0.03	0.68	−0.01	−0.06 to 0.04
	Education level					
	-Direct effect	1.11	0.68	0.10	0.12	−0.22 to 2.44
	-Indirect effect	0.37	0.32	0.24	0.04	0.25 to 0.99
	-Total effect	1.48	0.74	0.04	0.16	0.03 to 2.93
	Income					
	-Direct effect	1.42	1.36	0.29	0.09	−1.24 to 4.08
	-Indirect effect	2.18	0.72	0.00	0.12	0.77 to 3.59
	-Total effect	3.60	1.41	0.01	0.21	0.84 to 6.36
Physical QoL	Perceived Stress					
	-Direct effect	−0.17	0.04	0.001	−0.29	−0.24 to −0.10
	-Indirect effect	No path				
	-Total effect	−0.16	0.04	0.001	−0.28	−0.23 to −0.09
	Coping Strategies					
	-Direct effect	−0.03	0.02	0.13	−0.12	−0.06 to 0
	-Indirect effect	No path				
	-Total effect	−0.03	0.02	0.13	−0.12	−0.06 to 0
	Uncertainty in Illness					
	-Direct effect	−0.05	0.02	0.01	−0.22	−0.08 to −0.02
	-Indirect effect	−0.02	0.01	0.08	−0.09	−0.03 to −0.01
	-Total effect	−0.07	0.01	<0.001	−0.32	−0.08 to −0.06
	Age					
	-Direct effect	−0.08	0.02	0.00	−0.30	−0.11 to −0.05
	-Indirect effect	No path				
	-Total effect	−0.08	0.02	0.00	−0.30	−0.11 to −0.05
	Education level					
	-Direct effect	0.48	0.20	0.02	0.17	0.09 to 0.87
	-Indirect effect	0.11	0.09	0.23	0.04	−0.06 to 0.28
	-Total effect	0.59	0.22	0.00	0.21	0.16 to 1.02
	Income					
	-Direct effect	0.09	0.39	0.80	0.02	−0.67 to 0.85
	-Indirect effect	0.20	0.21	0.34	0.03	−0.21 to 0.61
	-Total effect	0.29	0.41	0.47	0.05	−0.51 to 1.09
Psychological QoL	Perceived Stress					
	-Direct effect	−0.05	0.03	0.11	−0.15	−0.10 to 0
	-Indirect effect	No path				
	-Total effect	−0.04	0.03	0.12	−0.14	−0.09 to 0.01
	Coping Strategies					
	-Direct effect	−0.01	0.01	0.40	−0.07	−0.02 to 0
	-Indirect effect	No path				
	-Total effect	−0.01	0.01	0.40	−0.07	−0.02 to 0
	Uncertainty in Illness					
	-Direct effect	0.00	0.01	0.99	0.00	−0.01 to 0.01
	-Indirect effect	No path				
	-Total effect	−0.00	0.01	0.58	−0.04	−0.01 to 0.01
	Age					
	-Direct effect	0.00	0.01	0.53	0.60	−0.01 to 0.01
	-Indirect effect	No path				
	-Total effect	0.00	0.01	0.48	0.07	−0.01 to 0.01
	Education level					
	-Direct effect	0.43	0.14	0.00	0.26	0.16 to 0.70
	-Indirect effect	0.00	0.03	0.86	0.00	−0.05 to 0.05
	-Total effect	0.43	0.14	0.00	0.26	0.16 to 0.70
	Income					
	-Direct effect	−0.02	0.28	0.93	−0.01	−0.56 to 0.52
	-Indirect effect	−0.02	0.09	0.84	−0.01	−0.19 to 0.15
	-Total effect	−0.04	0.26	0.87	−0.01	−0.54 to 0.46

QoL = Quality of life; SE = Standard error; Std. coef= Standardized coefficient; CI = Confidence interval

## Discussion

This study aimed to investigate the relationship between uncertainty in illness, perceived stress, coping strategies, and QoL in patients with CAD.

Poorer physical and psychological QoL was found among patients who had less than a Diploma education level (P = 0.008). Similarly, Mandal et al. found that patients with coronary heart disease and higher education levels reported a better QoL [43]. Individuals with higher education may have better access to health-related information, leading to improved health literacy compared to those with less educational background [44]. Specifically, inadequate health literacy can hinder patients from acquiring essential primary care skills, potentially resulting in negative disease outcomes [45]. Furthermore, studies have indicated that increased knowledge about the disease is associated with a health-promoting lifestyle and enhanced QoL [46]. Additionally, research by Soleimani et al. showed that participants with college or intermediate education levels were more likely to experience a higher QoL [47]. However, Medeiros et al. did not find a significant link between education level and QoL in cancer patients [48]. This difference may be due to the nature of the diseases studied, as cancer can adversely impact patients in ways that make education level a less reliable predictor of their QoL.

Patients who were divorced or widowed and living alone showed a poor physical QoL. Previous research has shown that personal characteristics, such as a sense of coherence (referring to the individual resources and strengths used to meet demands) [49], as well as support from spouses and family, are crucial for health-related QoL in patients with heart disease [50], [51]. In the study by Varghese and Kumar, 71.5% of patients with acute coronary syndrom were married, and the majority of them lived with family, which was associated with better QoL [52].

In the current study patients whose income surpassed their expenses reported a better physical QoL. If patients are financially supported by their family or government, they may be less worried about their healthcare and medical costs. Therefore, they may experience higher QoL [53]. Zuriati et al. found a positive significant relationship between income and physical QoL [54]. Najafi et al. found a significant association between high socioeconomic status and improved QoL in patients undergoing coronary artery bypass graft surgery [55]. This finding contrasts with the conclusions of Soleimani et al., who reported that patients with low and middle socioeconomic status often indicated a better QoL [47]. Additionally, Medeiros et al. could not establish a significant correlation between socioeconomic status and QoL among cancer patients [48]. These inconsistencies may be linked to the sanctions imposed on Iran in recent years by the international community. Such sanctions might have affected the general population by driving up economic inflation, increasing the cost of goods and energy, raising unemployment rates, and leading to shortages of essential supplies, including medications [56]. As a result, having a higher socioeconomic status may not necessarily ensure better access to care, treatment, medications, and medical services, and therefore, it may not enhance their physical QoL.

Based on the results of the sudy patients who had no history of dyslipidemia and had a shorter duration of illness reported a better physical QoL. The absence of dyslipidemia among patients with high physical QoL scores suggests that proactive management of chronic conditions may serve as a critical pathway for optimizing physical health. Dyslipidemia, characterized by abnormal lipid levels, is commonly associated with cardiovascular diseases and can significantly impact physical health. Patients without this condition likely have a lower risk of developing serious health issues, contributing to a more favorable physical state. A study by Zuriati et al. have shown that longer illness duration is associated with poorer physical QoL outcomes, as patients experience greater physical limitations and functional decline, highlighting the cumulative impact of chronic illness on physical health [54].

There was a negative corrolation between uncertainty in illness and physical QoL (r = −0.32, P < 0.001). Also, we found that uncertainty in illness have a direct effect on physical QoL (β = −0.05, Std. coef = −0.22, CI = −0.10 to 0, P = 0.01). In this study, the average score of uncertainty in illness was 66.77, which is similar to the study conducted in patients with chronic disease [25], [57]. Based on the results of a study by Zhang et al., an increased understanding the condition by patients can improve their QoL [58]. Guan et al. indicated that patients’ uncertainty in illness directly, and negatively influenced their physical QoL [25]. These findings further reinforce the link between uncertainty and adaptation outcomes, as suggested in Mishel’s uncertainty in illness theory [14]. Consistent with earlier studies [25], [57], our results offer evidence in favor of using uncertainty management interventions to enhance physical QoL for patients with CAD.

Patients who experienced greater uncertainty in illness reported higher levels of perceived stress (r = 0.50, P < 0.001) (β = 0.21, Std. coef = 0.50, CI = 0.17 to 0.24, P < 0.001). Uncertainty in illness regarding symptoms, treatment, and outcomes related to the disease is a primary source of stress in chronic illnesses [59]. Previous studies have also shown that uncertainty is a strong stressor for individuals [60], which aligns with the findings of the present study in patients with CAD. Kurita et al. also found that uncertainty in illness in lung cancer patients is positively associated with perceived stress [61]. Guan et al. noted that the diagnosis and treatment of a disease can be a stressful experience, with uncertainty in illness being a significant source of that stress [25].

In addition, the present study demonstrated that perceived stress has a negative correlation and direct effect on physical QoL (r = −0.37, P < 0.001) (β = −0.17, Std. coef = −0.29, CI = −0.24 to −0.10, P = 0.001), which aligns with the study by Endrighi et al. [21]. Furthermore, uncertainty in illness negatively predicts overall QoL in patients with kidney tumors and prostate cancer [62]. Therefore, healthcare providers, by taking appropriate measures to reduce patient stress, may improve the QoL of patients with CAD who are experiencing illness uncertainty.

Additionally, uncertainty in illness was negatively and significantly correlated with coping strategies (r = −0.41, P < 0.001). Uncertainty in illness also showed a direct negative effect on coping strategies (β = −0.31, Std. coef = −0.36, CI = −0.44 to −0.18, P < 0.001). Although the indirect pathway through coping strategies was not statistically significant, the direction of the association suggests that higher uncertainty may reduce patients’ ability to employ adaptive coping responses. According to Mishel’s uncertainty in illness theory, patients’ interpretation of uncertainty determines their adaptation process [14]. When uncertainty is perceived as a danger, ineffective coping responses may occur, whereas effective coping can facilitate adaptation and improve quality of life. Therefore, interventions that improve illness-related knowledge and strengthen coping skills may help patients manage uncertainty more effectively.

In the mediation model, some pathways demonstrated differences between direct and total effects, suggesting the possibility of inconsistent mediation or suppressor effects. For example, although uncertainty in illness had a significant direct association with physical QoL, the indirect pathway through perceived stress and coping strategies showed a different magnitude and direction. Such findings indicate that the relationship between illness uncertainty and QoL may involve complex psychological mechanisms rather than a single pathway. However, because the present study was cross-sectional, these findings should be interpreted as associations rather than causal effects. Future longitudinal studies are needed to clarify the temporal sequence of these relationships. These results support the stress and coping framework proposed by Lazarus and Folkman, suggesting that patients’ appraisal of illness-related uncertainty and their coping responses may influence adaptation and perceived quality of life. The indirect effect of uncertainty in illness on physical quality of life through perceived stress was not statistically significant (P = 0.08), indicating that perceived stress did not act as a significant mediator in this pathway. Similarly, the indirect pathway through coping strategies was not significant, although uncertainty in illness showed a significant direct negative association with coping strategies. This finding suggests that uncertainty may influence patients’ coping responses, but the extent to which coping strategies translate this effect into changes in quality of life requires further investigation. The absence of statistically significant indirect effects indicates that inconsistent mediation or suppressor effects should be interpreted cautiously. Although differences between direct and total effects were observed, the confidence intervals of the indirect effects included zero, and therefore no significant suppressor effect was confirmed. These findings are consistent with Mishel’s uncertainty in illness theory and Lazarus and Folkman’s stress and coping framework, which emphasize that patients’ appraisal of uncertainty and coping responses interact in complex ways during adaptation to chronic illness.

Psychological QoL showed no significant associations with illness uncertainty or perceived stress. The psychological QoL measure may have limited sensitivity to detect nuanced variations related to illness uncertainty and perceived stress in this population. Future work could incorporate multi-method assessments (e.g., clinical interviews, momentary assessments, or additional validated scales covering anxiety, depression, coping, and resilience) to capture broader psychological functioning. Cultural norms around expressing psychological distress, coping strategies, or illness-related concerns might attenuate observed associations. If the sample is culturally homogeneous or if cultural factors influence reporting patterns, effects on psychological QoL could be reduced or obscured. Examining moderators such as cultural values, social support, or stigma in future analyses could illuminate these dynamics. Psychological QoL may fluctuate with disease trajectory, treatment phases, or adaptation over time. A cross-sectional design may fail to capture associations that change over time. Because the data were collected at a single point in time, temporal sequences among the variables could not be established. Therefore, although path analysis can provide valuable insights into potential relationships and underlying mechanisms, causal interpretations should be made with caution. The observed associations suggest possible directions of influence rather than confirmed causal effects. Future studies employing longitudinal designs are needed to examine changes over time and to provide more robust evidence regarding the causal relationships proposed in the present model.

The sample is drawn from a single center and reflects specific eligibility criteria; the external validity may be limited to similar patient populations and settings. It is recommended to replicate in diverse settings, including multi-center samples and broader demographic groups, to assess the broader applicability of the findings. In addition, the generalizability of the findings may be limited, as the study sample was restricted to Iranian patients with coronary artery disease; therefore, the results should be interpreted with caution when applied to other populations or cultural contexts. Additionally, in this study, the coping strategies subscales were not included in the model. It seems that another study with a larger sample size is needed, and it is recommended that more confounding variables be included in the model in future studies.

Although several additional clinical and treatment-related confounding factors may influence quality of life in patients with CAD, it was not feasible to include all potential confounders because doing so would substantially increase the length and complexity of the demographic questionnaire. Therefore, the present study mainly focused on the principal psychosocial variables of interest. Future studies are recommended to include more detailed clinical and treatment-related variables.

### Clinical implications

This study highlights that illness uncertainty and perceived stress significantly affect physical quality of life in patients with coronary artery disease, suggesting the need for psychosocial interventions integrated into routine cardiac care. Structured patient education can reduce uncertainty by clarifying illness-related information, while cognitive-behavioral and mindfulness-based strategies can alleviate stress and improve emotional regulation. Coping-skills training that emphasizes problem-solving and adaptive coping may further enhance patients’ ability to manage their condition. Embedding these approaches within cardiac rehabilitation programs and promoting interdisciplinary collaboration between cardiology and mental health professionals can help translate these psychosocial pathways into improved clinical outcomes and overall quality of life. In summary, this study’s findings emphasize that optimizing CAD outcomes requires addressing both physiological and psychosocial dimensions of care. Reducing illness uncertainty and perceived stress through structured psychosocial management can not only improve patients’ physical functioning but also foster greater engagement in rehabilitation and adherence to long-term treatment plans. Further integration of the mediation findings indicates that interventions should not only target stress reduction but also address the underlying uncertainty appraisal process and coping responses. Tailored interventions may help patients develop more adaptive responses to illness-related uncertainty and potentially improve physical quality of life outcomes.

## Conclusions

Uncertainty in illness and perceived stress are key psychosocial factors influencing the quality of life of patients with CAD. To address these, healthcare providers should implement targeted interventions such as psychological counseling to manage illness-related fears, structured patient education programs to clarify disease information and reduce uncertainty, and stress management or coping-skills training (e.g., mindfulness or cognitive-behavioral approaches) to enhance emotional resilience. Incorporating family support and routine psychosocial screening into cardiac rehabilitation can further promote adaptation and improve both physical and psychological quality of life. Future longitudinal studies with larger and multi-center samples are recommended to examine temporal relationships among illness uncertainty, perceived stress, coping strategies, and quality of life and to provide stronger evidence regarding the causal pathways proposed in this model.

## Supporting information

S1 File STROBE checklist. (DOCX)

## Abbreviations

CAD: Coronary artery disease
QoL: Quality of life
